# European soybean to benefit people and the environment

**DOI:** 10.1038/s41598-024-57522-z

**Published:** 2024-03-31

**Authors:** Jose L. Rotundo, Rachel Marshall, Ryan McCormick, Sandra K. Truong, David Styles, Jose A. Gerde, Emmanuel Gonzalez-Escobar, Elizabete Carmo-Silva, Victoria Janes-Bassett, Jennifer Logue, Paolo Annicchiarico, Chris de Visser, Alice Dind, Ian C. Dodd, Louise Dye, Stephen P. Long, Marta S. Lopes, Joke Pannecoucque, Moritz Reckling, Jonathan Rushton, Nathaniel Schmid, Ian Shield, Marco Signor, Carlos D. Messina, Mariana C. Rufino

**Affiliations:** 1 Corteva Agriscience, Seville, Spain; 2 Lancaster Environment Centre, Lancaster University, Lancaster, UK; 3 Gro Intelligence, New York City, NY USA; 4 Corteva Agriscience, Johnston, USA; 5 School of Biological and Chemical Sciences, University of Galway, Galway, Ireland; 6 Instituto de Ciencias Agrarias de Rosario, UNR, CONICET, Zavalla, Argentina; 7 School of Environmental Sciences, University of Liverpool, Liverpool, UK; 8 Lancaster Medical School, Lancaster University, Lancaster, UK; 9 Council for Agricultural Research and Economics (CREA), Rome, Italy; 10 Wageningen University and Research, Wageningen, The Netherlands; 11 Research Institute of Organic Agriculture (FiBL), Frick, Switzerland; 12 School of Psychology and Food Science and Nutrition, University of Leeds, Leeds, UK; 13 Departments of Crop Sciences and of Plant Biology, University of Illinois, Champaign, USA; 14 Sustainable Field Crops, Institute of Agrifood Research and Technology (IRTA), Lleida, Spain; 15 Flanders Research Institute for Agriculture, Fisheries and Food (ILVO), Merelbeke, Belgium; 16 Leibniz Centre for Agricultural Landscape Research (ZALF), Müncheberg, Germany; 17 Department of Crop Production Ecology, Swedish University of Agricultural Sciences (SLU), Uppsala, Sweden; 18 Centre of Excellence for Sustainable Food Systems, University of Liverpool, Liverpool, UK; 19 Rothamsted Research, Harpenden, UK; 20 Regional Agency for Rural Development (ERSA), Gorizia, Italy; 21 University of Florida, Gainesville, USA; 22 School of Life Sciences, Technical University of Munich, München, Germany

**Keywords:** Plant sciences, Plant breeding

## Abstract

Europe imports large amounts of soybean that are predominantly used for livestock feed, mainly sourced from Brazil, USA and Argentina. In addition, the demand for GM-free soybean for human consumption is project to increase. Soybean has higher protein quality and digestibility than other legumes, along with high concentrations of isoflavones, phytosterols and minerals that enhance the nutritional value as a human food ingredient. Here, we examine the potential to increase soybean production across Europe for livestock feed and direct human consumption, and review possible effects on the environment and human health. Simulations and field data indicate rainfed soybean yields of 3.1 ± 1.2 t ha^−1^ from southern UK through to southern Europe (compared to a 3.5 t ha^−1^ average from North America). Drought-prone southern regions and cooler northern regions require breeding to incorporate stress-tolerance traits. Literature synthesized in this work evidenced soybean properties important to human nutrition, health, and traits related to food processing compared to alternative protein sources. While acknowledging the uncertainties inherent in any modelling exercise, our findings suggest that further integrating soybean into European agriculture could reduce GHG emissions by 37–291 Mt CO_2e_ year^−1^ and fertiliser N use by 0.6–1.2 Mt year^−1^, concurrently improving human health and nutrition.

## Introduction

Soybean is the largest source of vegetable protein for animal worldwide and it is an important component of human diets in some areas of the world. The EU-27 + UK imports 33 M tonnes of soybean products annually at a cost of €14bn^1^. Soybeans have higher protein, poly- and mono-unsaturated fat, and lower carbohydrate content than other legumes^2^. Clinical studies indicate that soybean consumption benefits human health by lowering low-density-lipoprotein (LDL)-cholesterol concentrations^3^ and blood pressure compared to other legume grains^4^. High soybean diets are associated with lower the incidence of heart attack and stroke, with variation across study populations^5^, although protein replacement resulting in lower meat consumption might also play a role. Whilst other grain legumes may provide these benefits and are important for diversification of the system, soybean has potential to be the most productive, requiring fewer hectares per unit of product^6^. Global average dry bean yields (1980–2020) rose from 0.5 to 0.8 t ha^−1^ (60%), dry peas 1.3 to 2.0 t ha^−1^ (53%) and soybean 1.6 to 2.8 t ha^−1^ (74%)^7^. Historically, soybean yield increases over the years have been associated with both genetic progress and improved crop husbandry^8^. The increase in soybean yield associated with genetic progress is not related to incorporating genetically modified (GM) defensive traits, since all the genetic gain studies are conducted in the absence of pests and weeds^9^. Soybean was one of the first crop species to be fully sequenced, enabling adaptive breeding to different environments and to accelerate crop improvement^10–12^. Although soybean is higher yielding with a faster pace of yield improvement than other grain legumes, its cultivation has been associated with a large carbon footprint due to deforestation and negative biodiversity impacts^13^. The current soybean value chain configuration of the European food system and agricultural policies inhibit harnessing the benefits of greater human soybean consumption^14^.

European soybean yields are low in comparison to this crop’s potential^14,15^. Suboptimal air temperatures for growth and development likely limit soybean production in northern Europe by delaying early vegetative growth, causing flower and pod abscission, poor grain filling, and high moisture for harvest^16^. Low soil temperatures limit root hydraulic conductance and decrease leaf turgor and expansion whilst high irradiance can cause photoinhibition^17^. Soil water deficits constrain soybean yields in central and southern Europe^18^. Leaf expansion and photosynthesis vary among soybean genotypes, suggesting genotypes better suited to the diversity of European environments could be selected^19^. Many areas are well suited to soybean cultivation, giving immediate potential for expansion. A comprehensive European soybean trial network has the potential to supply essential data for effective adaptation to diverse environments and the identification of high-yielding varieties suitable for different regions.

European farmers favour cereal production over legumes partly because of their higher yields, profitability^20^, yield stability^15^, well-developed markets, and ineffective incentives for legume production^20,21^. Nitrogen (N) fixation in European soybean cultivation would reduce fertiliser use^22^, as soybean is typically cultivated without adding N fertiliser, benefitting farmers in regions that restrict N use to mitigate water pollution and avoiding impacts of high fertiliser costs^23^. Production within Europe would also address regulatory constraints on genetically modified (GM) foods and consumer preferences^24^; the EU aims to transition to more locally-produced protein-rich plant-based proteins^1^. This paper aims to critically assess the evidence and prospects for increased soybean production by identifying suitable regions within Europe for soy production whilst analysing opportunities for adaptation and the likely environmental benefits of alternative scenarios. We also review health and nutritional factors associated with consuming soy foods, identifying specific traits desirable to the food industry.

## Results

### Increasing soybean production in Europe

**Figure 1:**
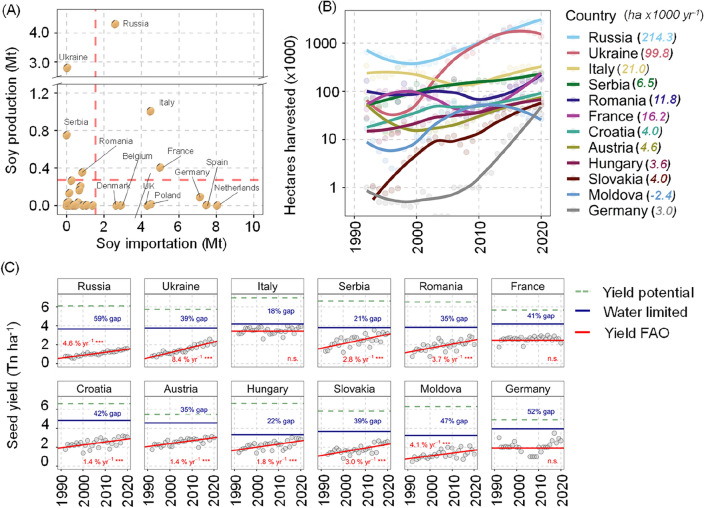
European soy production, imports, cultivated area and yield gaps. (**A**) Soybean seed production and import of soybean seed equivalents in millions of tons (Mt) for 2020 in 39 countries (EU-27 + UK plus 12 neighbouring European countries). Vertical and horizontal red dashed lines indicate mean imports and production. Countries above these averages are labelled. (**B**) Change in soy cultivated area (1992–2020) of 12 European countries that cultivate > 20,000 hectares since 2017. Numbers in parenthesis on the legend indicate the slope in thousand hectares per year for the period 2010–2020 (all linear models *p* < 0.05) (**C**) Change in soybean yield from 1992 to 2020 (grey dots). Red lines show the linear trend with the increase in yield as percentage per year (*p < 0.05, **p < 0.01, ***p < 0.001). Blue lines indicate simulated water-limited yield (mean of 20-year weather) and percent yield gap calculated as: (water-limited yield − observed yield in 2020)/water-limited yield. Green dashed lines indicate irrigated potential yield. All data except simulations are from FAOSTAT.

In year 2020, EU-27 + UK cultivated about 0.9 M hectares of soybeans to produce 2.7 M tonnes, approximately 8% of the total imports^7^; this area is projected to increase as demand for GM-free soybean for human consumption increases^1^. Nine countries account for ~ 85% of total EU-27 + UK imports (Fig. 1a). Except for Italy, these countries do not produce significant amounts of soybean, with eight of such countries (Netherlands, Germany, France, Poland, UK, Belgium, Denmark, and Ireland) located at the northern edge of current soybean cultivation. Soybean acreage, whilst still small relative to consumption, has significantly expanded in the last decade (Fig. 1b), notably in Italy, France, and Romania, currently at 12,000–21,000 ha year^−1^. Russia and Ukraine, non-EU countries with a tradition of soybean cultivation, substantially increased acreage by 214,000 and 99,800 ha year^−1^ to 2.7 and 1.36 Mha between 2010 and 2020, although the Russia-Ukraine war is expected to sharply decrease production and export, with 2022 official statistics pending. Comparing current yields with yield potential for 12 countries (with soy cultivation exceeding 20,000 ha) shows untapped opportunities to increase yields through improved agronomy (Fig. 1c), and improved cultivars. Nine of these 12 countries show a water-limited yield gap larger than 20%, and five larger than 40%. There are clear opportunities to close yield gaps in countries where no sustained yield gains have been observed in the last two decades (e.g. France, Italy and Germany).

**Figure 2:**
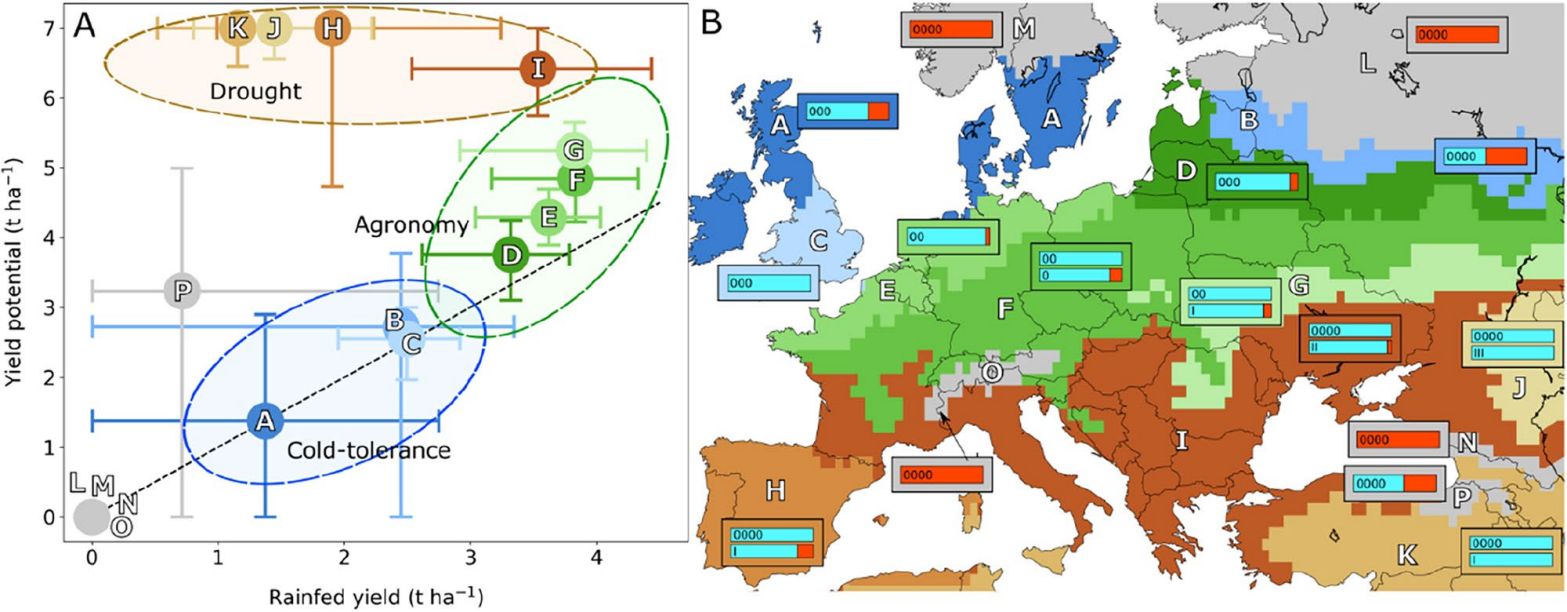
Soybean crop performance across Europe and its potential growing regions (16 clusters with similar outcomes for maturity groups 0000 to III). Clusters L to P were shaded dark grey because soy cannot complete its growth cycle in these regions. (**A**) Median and interquartile ranges of potential (y-axis) and rainfed yields (x-axis) for the maturity group with the largest yield within each region. Interquartile ranges for yields originate from temporal and spatial variation in weather, site-to-site variation in soil properties, and uncertainty associated with satellite-derived soil water initial conditions. Regions were assigned to crop improvement strategies: breeding for cold-tolerance in cool climates (blue shade), agronomic optimisation (green shade), and improving drought tolerance (brown shade); dashed line is the 1:1 regression line. (**B**) Geographical extent of each cluster; a bar plot inset is labelled with maturity group with the largest median rainfed and/or potential yield, and the bar is coloured cyan according to the proportion of years the maturity group successfully reached R7. For insets with two bars, the upper bar represents the maturity group with the largest median rainfed yield, and the lower bar represents the maturity group with the largest median yield potential. Insets with one bar indicate the same maturity group had the largest median potential and rainfed yields. The proportion of years failing to achieve R7 is coloured red, and the proportion of years with crop harvest success are coloured in light blue. The map was created using Python 3.10.0 (https://www.python.org/downloads/).

Crop modelling shows a large area (clusters D, E, F, and G, Fig. 2) of 70 M hectares from continental western, through central to eastern Europe where economic yields of soy (> 3 t ha^−1^) are feasible under rainfed conditions with a crop success rate > 85% and where better agronomic practices would close a yield gap of about 1 t ha^−1^ (Table 1, Fig. 1c). These practices involve weed control, reducing pest and disease incidence, managing planting density and row spacing, and selecting cultivars adapted to local environments, tolerating cold stress, in-season dry spells, and humid conditions during dry-down. These simulations show that improved agronomy could allow maturity groups between I and 000 to reach their yield potential. South of this optimal region are the Mediterranean and Balkan regions (cluster I, 56 Mha), with a larger yield gap (> 2 t ha^−1^) related to water deficits. Breeding for drought tolerance and adjusting maturity group could also benefit large areas of western Asia such as most of Türkiye (cluster K) and Kazakhstan (cluster J). Cluster H (Spain and Portugal) includes marginal agroecologies for soybean, where production currently requires irrigation. Cool temperatures and a shorter growing season in north-eastern Europe and southern UK (clusters B and C), limit potential yields to < 3 t ha^−1^; overcoming cold-tolerance requires breeding investments to identify phenotypes adapted to low soil temperature at sowing time and ability to develop photosynthetically competent leaves in chilling spring temperatures. A temperature-sensitive polymer seed coat is successfully used in the US Midwest to prevent seed and seedling damage during cold events following early season sowing^25^. Early spring air temperatures of 8–10 °C cause photodamage^26^ and slow crop development by limiting leaf expansion thereby compromising radiation capture, leading to crops failing to reach maturity or achieving low yields (< 1.5 t ha^−1^) in northern UK, Ireland and Scandinavia (cluster A). Current soybean cultivars cannot establish in the coldest environments of mountain and sub-arctic regions (clusters L, M, N and O, Table 1).

**Table 1:** Description of clusters and associated regions based on similar simulated soybean performance (potential and water-limited yields, and median crop success rate) for maturity groups from 0000 to III. Clusters L-P shaded grey represent the clusters where soy cannot complete its cycle under current climate.

Cluster	Region name	Climate	Current land use % of arable (top 3 crops by area)	Arable land (ha)	Land area as arable (%)	Yield potential ( t ha^−1^)	Rainfed yield ( t ha^−1^)	Crop success rainfed (%)	Maturity group potential yield	Maturity group rainfed yield
A	Northern Europe	Temperate maritime	Wheat (37%), barley (31%), rapeseed (10%)	6,124,324	18.1	1.6 ± 1.5	1.5 ± 1.3	75	000	000
B	North-eastern Europe	Continental	Wheat (45%), barley (13%), sunflower (8%)	2,247,233	9.1	2.0 ± 1.9	1.8 ± 1.6	50	0000	0000
C	Southern UK	Maritime	Wheat (41%), barley (26%), rapeseed (12%)	4,646,324	34.7	2.5 ± 0.8	2.4 ± 0.7	100	000	000
D	North-eastern Europe	Humid continental	Wheat (38%), barley (11%), rapeseed (11%)	6,976,458	18.4	3.3 ± 1.5	2.9 ± 1.4	90	000	000
E	Western Europe	Temperate maritime	Wheat (35%), barley (15%), rapeseed (8%)	13,225,677	39.4	4.1 ± 1.2	3.4 ± 1.1	95	00	00
F	West to eastern Europe	Continental to intermediate	Wheat (31%), barley (13%), maize (10%)	37,826,068	28.5	4.1 ± 1.4	3.6 ± 1.1	85	0	00
G	East Europe	Humid continental	Wheat (27%), maize (20%), sunflower (20%)	13,063,593	31.4	4.6 ± 1.9	3.6 ± 1.1	90	I	00
H	Southern Europe	Mediterranean hot summer	Olives (22%), barley (20%), wheat (15%)	12,487,126	18.6	5.1 ± 2.9	2.2 ± 1.5	80	I	0000
I	Southern Europe	Mediterranean humid continental	Wheat (29%), maize (18%), sunflower (13%)	55,649,190	25.8	5.8 ± 1.9	3.4 ± 1.3	95	II	0000
J	Central Asia	Cold semi-arid	Wheat (48%), barley (14%), sunflower (11%)	1,754,289	7.8	6.7 ± 0.6	1.6 ± 0.9	100	III	0000
K	West Asia	Dry summer subtropical	Wheat (37%), barley (17%), olives (7%)	18,165,133	19.6	6.2 ± 1.9	1.5 ± 1.3	100	I	0000
*L*	*North-eastern Europe*	*Sub-arctic*	*Wheat (41%), barley (16%), sunflower (11%)*	*5,412,229*	*8.0*	*0.2* ± *0.8*	*0.2* ± *0.7*	*0*	*na*	*na*
*M*	*Northern Europe*	*Maritime sub-arctic*	*Wheat (35%), barley (31%), oats (14%)*	*729,597*	*4.2*	*0.2* ± *0.7*	*0.2* ± *0.7*	*0*	*na*	*na*
*N*	*Southwest Russia*	*Hot summer continental*	*Wheat (33%), barley (12%), maize (10%)*	*480,846*	*6.6*	*0.8* ± *1.8*	*0.7* ± *1.5*	*0*	*na*	*na*
*O*	*Alps*	*Alpine*	*Wheat (31%), maize (12%), barley (10%)*	*899,790*	*9.3*	*0.3* ± *1.0*	*0.3* ± *1.0*	*0*	*na*	*na*
*P*	*Asia Southeast Black Sea*	*Humid*	*Wheat (36%), barley (16%), olives (4%)*	*1,387,943*	*20.4*	*2.6* ± *2.4*	*1.4* ± *1.5*	*60*	*0000*	*0000*

Significant values are in italics.

### Environmental sustainability

Alternative scenarios representing agronomic potential for European soybean expansion show significant prospective environmental benefits. First, taking hypothetical scenarios that avoid displacing existing crop cultivation, planting half of set-aside area^27^ in appropriate agroclimatic regions, soybean would produce 9–13 Mt soy annually at current (Scenario 1, Sc-1) or potential (Sc-2) yields (Table 2). This would replace up to 40% of current EU imports, avoiding 13–19 Mt CO_2_ equivalent (CO_2_e) from soybean production and transport from Brazil, plus an avoided carbon opportunity cost (COC) of 24–35 Mt CO_2_e by sparing 1.8–2.7 Mha of soy cultivation in Brazil. Alternatively, diversifying crop rotations by replacing 10% of current EU wheat cultivation (in areas where soy could yield ≥ 3 t ha^−1^) with soybean cultivation, could produce 11–23 Mt soybean at current (Sc-3) or potential (Sc-4) yields, avoiding 16–31 Mt CO_2_e annually from imported soybean. However, based on average European wheat yield based on Agribylase LCA data for compensatory wheat production, this would displace 26–34 Mt wheat production, requiring a net increase in global cropland area of ca 1 Mha in Sc-3 as weighted-average annual soybean yields in Europe (3.2 t ha^−1^) are lower than Brazil’s (ca 5 t ha^−1^). A COC of 13 Mt CO_2_e would negate over 80% of the production and transport GHG saving (Table 2). Linking soy production with diet shifts, scenarios 5 and 6 indicate large area (6.5–15 Mha), fertiliser-N (0.6–1.2 Mt N year^−1^), and production-related GHG (47–93 Mt CO_2_e year^−1^) savings by substituting chicken, pork, and milk with half of the soybean produced on 10% of land currently used to cultivate wheat, and where high soy yields (≥ 3 t ha^−1^) can be achieved. On a protein basis, soy produced in these scenarios could substitute 30–58% of chicken consumption and 8–16% of pork consumption, alongside substitution of 8–16% of milk consumption on a volume basis across the EU^28^. Including COC, these scenarios 5 and 6 could mitigate up to 291 Mt CO_2_e year^−1^. The area planted with soybean (3.6–4.8 Mha) would be only slightly larger than projected plantings with soybean and pulses (3.4 Mha) over the coming years^27^.

**Table 2:** New and avoided crop production, and associated area and fertiliser nitrogen (N) application changes, associated with six scenarios of soybean cultivation across agroclimatic regions and fractions of areas indicated in Table 1. Also shown are net production and carbon opportunity cost greenhouse gas emissions savings associated with each of the scenarios, including avoided animal production (and associated animal feed crop production). Sc-1 and Sc-2 represent soybean cultivation on 50% of EU set-aside at current or potential yields from Table 1, respectively. Sc-3 and Sc-4 represent replacement of 10% of wheat cultivation (by area) with soy cultivation in regions where soybean yields of at least 3 t ha^−1^ could be achieved, at current or potential yields, respectively. Sc-5 and Sc-6 are derivatives of Sc-3 and Sc-4, assuming that half of the European soybean production displaces animal protein, equally split across chicken, pork and milk.

Scenario	European soybean	Imported soybean	Displaced wheat	Barley feed	Maize feed	Rapeseed feed	Sunflower feed	Cropland area	Fertiliser-N	Production GHG	Carbon opportunity cost
	Mt year^−1^							Mha	Mt year^−1^	Mt CO_2_e year^−1^	
Sc-1	9	− 9	0					− 1.8		− 13	− 24
Sc-2	13	− 13	0					− 2.7		− 19	− 35
Sc-3	11	− 11	26					1.0		− 16	13
Sc-4	23	− 23	34					− 0.1		− 31	− 2
Sc-5	11	− 16	13	− 0.7	− 42	− 0.8	− 0.3	− 6.5	− 0.6	− 47	− 86
Sc-6	23	− 31	10	− 1.3	− 83	− 1.5	− 0.6	− 15.0	− 1.2	− 93	− 198

### Soybean for nutrition

**Figure 3:**
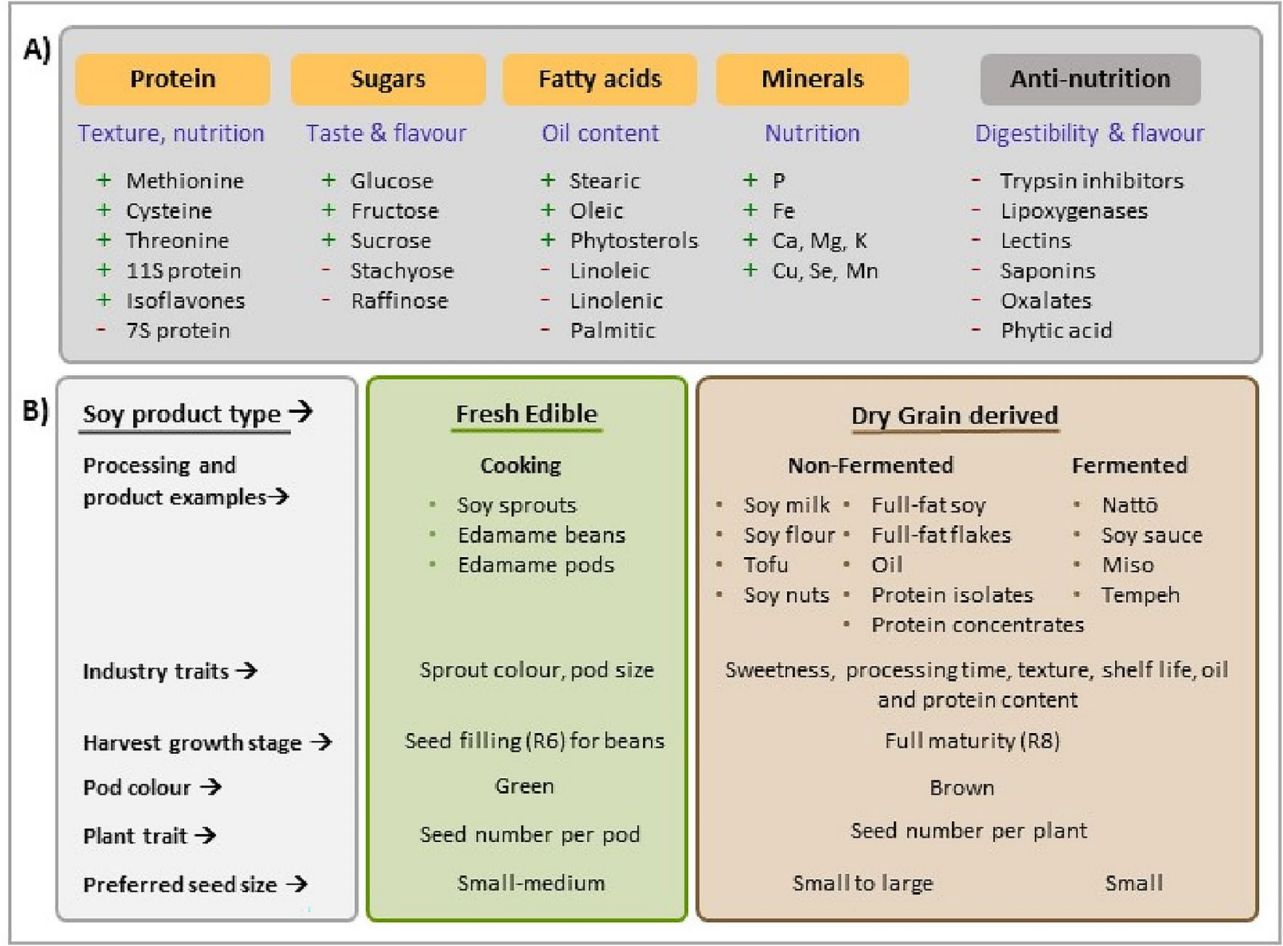
Soybean and soy-product traits, qualities, and breeding aims: (**A**) Crop traits for soy recommended to increase (green plus symbol) or to decrease (red minus symbol). The grey box indicates negative selection for anti-nutritional compounds and the orange boxes indicate positive selection of traits (protein, sugars, fatty acids and minerals). (**B**) Soy products from fresh edible and mature grain and target traits for crop improvement contributing to better food and feed quality.

The large soybean import demand of the EU would be mitigated if more soybean was used for direct human consumption rather than to feed livestock. Here we review reasons and opportunities for such a shift. Studies indicate that soy-based foods provide simultaneously protein, essential fatty acids, carbohydrates, dietary fibre, minerals and vitamins and beneficial phytochemicals such as isoflavones and phytosterols (Supplemental Table 1). Most soybean imported into Europe is currently used as livestock feed, with soybean breeding aiming to increase yield instead of enhancing soy functional properties^29^. Processing improves quality of feed- and food-grade soybean by reducing the concentrations of anti-nutritional compounds for humans and livestock, such as trypsin inhibitors, allergens, saponins, lectins, phytate, and the raffinose family oligosaccharides (RFO)^30^, justifying common breeding goals with specific targets for fresh edible and mature grain products (Fig. 3).

Soybeans are rich in essential amino acids, especially lysine. When considering protein quality, soybeans score almost as high as milk proteins and well above proteins from other legume or cereal sources^31^. The limiting amino acids to fully meet human and monogastric requirements are the sulphur-containing ones: methionine and cysteine^30^. There is genetic diversity in cysteine and methionine in storage proteins, glycinin (11S) and β-conglycinin (7S), with 11S having up to 4 × higher concentration of these amino acids than the 7S protein^32^. Increasing the 11S/7S ratio improves soy quality, with enhanced texture, chewiness, springiness, and hardness of tofu^33^. Modifying soybean amino acid profile has seen limited success, as high-throughput germplasm screening has not been cost-effective and bioengineering is unlikely to be accepted in the EU^34^. Genotype and growing regions affect seed protein concentration, and high protein concentration is usually associated with lower yields^35^, requiring simultaneous screening of traits. However, a wide range of protein, oil and mineral composition exists within soybean germplasm collections, providing clear breeding opportunities to deliver cultivars to meet nutritional requirements^36^.

Compared to other legumes and cereals, soybeans have more diverse nutritional properties and higher concentrations of minerals (potassium, calcium, magnesium, iron and zinc) and B vitamins (Supplementary Table 1). Riboflavin, vitamin A and vitamin E, whilst not in high concentrations, enhance the nutritional profile of soy foods^37^. Certain genotypes and environments can enrich key minerals for human nutrition such as selenium, copper, iron, manganese and zinc in soy^38^. For some minerals, most notably calcium and iron, soy has higher bioavailability than other legumes^39^. Soy’s high iron concentrations can help raise iron levels in deficient populations; the ferritin form of iron found in soy has greater bioavailability than the phytate-complexed form found in cereal grains^40^. Increased soybean iron concentration in varieties with higher nodulation^41^, could help improve iron status in humans^42^.

Anti-nutritional compounds associated with soy proteins such as trypsin inhibitors reduce protein digestibility in the human gut^43^ and in monogastric livestock^44^ or inhibit nutrient and vitamin uptake resulting in deficiencies^30^. Fermentation (for miso and tempeh), soaking (tofu) and cooking can reduce anti-nutritional effects for humans^30^ and thermal processing reduces trypsin inhibitors activity in livestock feed although protein denaturation reduces digestibility^30^. Genetic variability in trypsin inhibitor, phytate and tannin content^45^ could be exploited by using molecular methods to reduce concentrations of these compounds^43^. Thermal processing inactivates carbohydrate-binding proteins such as lectins^44^, but heat-resistant RFOs such as stachyose and raffinose may remain^43^. RFOs are substrates for microbial fermentation in the human gut, causing bowel discomfort^46^. RFOs reduce feed digestibility for monogastric animals^47^, although they ferment into fatty acids in pig intestines showing prebiotic properties^48^. Soy breeding aims to reduce RFOs concentrations whilst increasing total sugars^49^ since high sucrose, fructose and glucose increase the sweetness of soymilk, tofu and natto^46,50, 51^.

Soybeans are classified as allergenic foods, however, the prevalence of this allergenicity among the population with food allergies is ~ 3%, while milk, peanuts, tree nuts, eggs, and shellfish account for ~ 75%^52^. At least 16 protein fractions in soybeans are considered allergens^53^. This makes it difficult to tackle this issue solely through plant breeding, therefore additional processing strategies are being explored to reduce soybean allergenicity through thermal, ultrasound, and cold plasma treatments, enzymatic hydrolysis, and chemical modifications^54^.

### Soybean and health

Consuming soy foods can lower cholesterol concentrations by displacing high-saturated fat animal proteins in human diets^3^. A meta-analysis of 46 studies indicated soy protein consumption decreased cholesterol by 3–4% more than with other non-soybean protein sources^55,56^. The mechanisms by which soy reduces LDL-cholesterol are not well understood and may relate to the 7S globulin upregulation of LDL receptor^57,58^, the effect of isoflavones^59^, or phytosterols which can reduce cholesterol absorption^60^. Isoflavones are associated with 7S and mimic the effects of estrogen in the human body^61^. Their moderate consumption is associated with reduced risk of heart disease and hormone-related cancers, and reduction in adverse effects of hormone imbalances^62^. Soy has higher isoflavone concentrations than pulses, with sowing date, density^63^ and cultivar^64^ promising further enhancement. Seed phytosterol concentrations vary between 202 and 843 µg g^−1^ and positively correlate with oil content^64^, and phytosterols can be extracted for dietary supplements.

Soy oil has a relatively high ratio of linoleic to α-linolenic (ca. 7:1)^65^ and efforts to change it are currently undergoing^66^. Since linoleic is inflammatory and α-linolenic is anti-inflammatory, they should be consumed at a ratio of 1:1–2:1 (linoleic:α-linolenic)^67^. Research showed that reducing the α-linolenic and saturated fats does not affect the concentrations of beneficial phytochemicals and antioxidants^68^. Recent discoveries on the function of key genes in fatty acids synthesis can be used to improve soy oil profile^69^. Concentrations of oleic acid higher than 70% and concentrations of α-linolenic lower than 3% improve oil stability and provide health benefits as food and feed^70,71^. Soybean breeders have developed cultivars with modified fatty acid compositions targeted to increase the oxidative stability and the improvement of other technological properties such as plasticity or melting temperature through the reduction of the concentration of polyunsaturated fatty acids (linoleic and α-linolenic)^66^.

### Soybean comparison to other protein sources

Soybean has one of the highest concentrations of protein among grain legumes and cereals, making it a suitable feedstock to produce substitutes for animal-sourced foods such as tofu, soy milk, edamame, tempeh, and, increasingly, textured vegetable protein (meat analogue). The best quality soybean varieties produce about 40–50% protein, 20–25% total edible oil and 15% soluble carbohydrates^72^. Soybeans provide a higher ratio of protein to saturated fat than animal-based products and a ca. 4 × higher protein to carbohydrate ratio than cereals (Supplementary Table 1). Although soy has lower sulphur amino acid (methionine and cysteine) content than cow milk, the protein quality of soy milk is higher than that of other plant-based milk substitutes such as almond, rice, coconut^73^. Soy is also the main source of protein for pigs^74^, and of lysine to both pigs and poultry^20^; improving the amino acid profile of soy could benefit the livestock industry. Whilst plant proteins generally have lower digestibility than animal proteins^75^, soy has the highest digestibility among legumes with 80% for soybean and > 95% for soy protein isolate^76^, and therefore it is a preferred ingredient for meat substitutes in human diets.

### Soybean traits for food use

Nutritional and industrial properties of food-grade soy vary with plant developmental stages (green *versus* mature beans), with processing, and different soy products. When producing soybean sprouts, the light used for germination can promote chlorophyll formation affecting nutritional quality and marketability^77^. Sprouting decreases the activity of hemagglutinin, lipoxygenases, and trypsin inhibitors that impair protein digestibility. Seed concentrations of bitter-tasting aglycones, which regulate the flavour of sprouts decrease in partially shaded maize-soybean intercropping^78^. At the green picking stage, edible pods (consumed as Edamame) are rich in vitamins B1 and B2, iron, calcium, phosphorus, and protein content, but low in lipoxygenases, which increase in concentration with pod maturity^79^. Water deficit during seed filling increases protein concentration but decreases seed size^80^. Time to pod maturity influences glucose content, seed colour, seed texture, seed size and quality^81^, with essential amino acid concentrations increasing in mature beans^1^. Pod harvest time is critical for quality and nutritional properties of fresh edible soy.

Soaking soybeans before processing is common practice to soften the beans and reduce cooking time for human consumption. However, soaking can leach beneficial water-soluble antioxidants into water^82^. Cooking can decrease concentrations of other health-promoting compounds such as flavonoids and phenolics by 68–78% and 43–62% respectively^82^. Larger seeds reduce the negative effects of soaking, with 5.5–7.3 mm seeds preferred for natto production for their higher water absorption capacity and rapid fermentation, which retain flavour^50^. Therefore, improvement in soybean properties and size can help improve specific production efficiencies.

## Discussion

Europe has historically had a deficit in plant-based protein sources, relying on imports from the Americas to feed livestock^1^. European aspirations to increase protein production to shorten agri-food supply chains, reduce the demand for imported protein rich feedstocks, and to supply the burgeoning market of plant-based products have become more acute with the Russia-Ukraine war leading to urgent calls to develop a comprehensive European protein strategy^83^. In 2018, The European Commission suggested avenues to increase domestic soybean production, including (i) R&D investment to increase yields via extension, training, and dedicated breeding programmes, (ii) additional subsidies for protein-rich crops, and ecological focus areas (EFA) for nitrogen fixing crops, (iii) phasing-out import of oil palm to increase the demand for soy oil and (iv) raising awareness about the benefits of soybean based products to change consumer behaviour, and to increase consumption of plant-based products^84^. Economic modelling of the first three strategies concluded that the most effective policies would support an annual increase in yields of 1% at EU level for 2020–2030^84^. Several European countries have observed these yield gains (Fig. 1c), and Eastern European climate and soils (similar to those of the US Midwest) should allow high yields (> 5 t ha^−1^)^14^.

Climate change is expected to increase the frequency of severe drought and heat waves in southern Europe, whilst northern areas may benefit from higher late season temperatures^85^. Drought is expected to constrain soybean production more than heat stress^86^ yet expansion of production areas as a result of temperature and CO_2_ effects may overcompensate yield reductions due to drought^87^. Four strategies are proposed here to grow soybean under future climate: (i) selecting for cold-tolerance of early growth and higher photosynthetic efficiency in maturity groups ranging from 0 to 0000 to adapt to the diversity of environments of a large area (clusters A, B, C, D and E) becoming increasingly warmer and some areas with sub-optimal radiation (clusters A and C); (ii) early-sowing of long-cycle cultivars (groups I to III) to escape end-of-season water deficit^88^ (clusters I, J, and K); (iii) relay and double-cropping cereals and soy to better utilise land and the thermal-time window in central Europe (clusters E, F and G); (iv) selecting drought tolerant cultivars for south-European environments especially cluster I, and cluster H where double cropping can be practiced. Suitability of variety selection by location is therefore crucial for expansion of soybean^89^.

To ensure that soy cultivars produce > 3 t ha^−1^ with a failure rate < 10% under future warmer and drier climates, a systematic assessment of genotypes is required. Diversity panels could be screened to identify drought and cold tolerance in soybean cultivars^12^. Limited variation in drought tolerance is reported in modern cultivars probably because of breeding in favourable environments^90^. However, genes associated with drought tolerance have been identified^91^ and genomic selection can effectively and efficiently select for drought tolerance by exploiting gene-marker linkages over several parts of the genome^92^. Projections indicate that Europe could achieve 50% self-sufficiency in soy production by further integrating soy into crop rotations^93^. Relay intercropping of cereals and legumes provides another option in specific conditions, with increased land use equivalent ratio probably derived from increased nutrient use efficiency^94^. This could help increase biodiversity and reduce nutrient surpluses^95^ although the practice needs optimisation and specialised machinery^96^.

Reducing animal feed demand (where soy production and consumption is linked with a diet shift away from meat) would reduce displacement of other crops in Europe, and spare significant areas of cropland overall, so that an additional 86–198 Mt CO_2_e year^−1^ could be sequestered via carbon farming activities (e.g. afforestation) on spared land^14,97^. Thus, European soybean cultivation could mitigate substantial quantities of GHG emissions when planted on set aside or ecological focus areas, or when cultivated within a food system that replaces crop-fed animal protein with plant protein. Combining animal protein substitution with carbon farming activities on spared land, could save 291 Mt CO_2_e annually.

Increasing soybean foods in human diets can improve human health by reducing LDL-cholesterol concentrations, blood pressure, the risk of cardiovascular events and supplementing iron-deficient populations. Increasing soy production in Europe is feasible, bringing additional benefits by greater alternatives for crop rotation, stability and food system self-sufficiency, and more sustainable farming by reducing negative impacts associated with intensive fertiliser use. Further, creating awareness of soybean health benefits needs to be accompanied by market development because the food industry has specific requirements to process soy such as protein concentration, oil quality and the reduction of anti-nutritional compounds.

The market for plant-based proteins is growing rapidly^1^, with most hot and cold meat replacements comprising soybean^98^, and soy milk having a higher protein content than other plant-based milk alternatives^99^. The increased demand for soy and the feasibility of increasing its production in Europe justify crop and agronomic improvement programmes that concurrently achieve higher productivity and better quality to deliver plant-based protein. Value chains based on European soy must allow cultivars and husbandry methods that meet industry and consumer demand. This appears to be critical as food-grade soy requires more flexible approaches than those currently on offer from exporting countries dependent on large-scale infrastructure and shipping. Large amounts of premium quality soybeans are required to achieve a dietary transition towards more climate-friendly foods.

### Methods

### Assessing European soy production and cultivated area

Data from EUROSTAT at NUTS 2 level was used to estimate 2020 area of arable land use and soy production per region^100^. As EUROSTAT data covers only EU nations, this data was supplemented by FAOSTAT country level data^7^ for the remaining model-simulated areas. Spatial analysis of the data was conducted in R using the ‘sf’ package^101,102^. Area of each NUTS 2 region/country per model simulated region was calculated using spatial intersection. The area of arable land/soy production per model simulated region was estimated using the fractional area of each NUTS 2 region/country within each model simulated region. Predominant arable crops for each model-simulated region were calculated in the same way, using FAOSTAT 2020 data only (due to differences in reporting of arable crop types between the two data sources).

### European soybean production and imports

Soybean “Area harvested”, “Yield”, and “Production quantity” was downloaded from FAOSTAT^7^ under the “Production”, “Crops and livestock” domains. Country filter was “Europe > (List)”, and Items were “Crops Primary”, “Soybeans” and years from 1992 to 2020 (29 years). The list of 24 countries with 29 years of records for these variables were: Albania, Austria, Bosnia, Bulgaria, Croatia, Czechia, France, Germany, Greece, Hungary, Italy, Latvia, Moldova, Netherlands, North Macedonia, Poland, Romania, Russia, Serbia, Slovakia, Slovenia, Spain, Switzerland and Ukraine. Analysis of soybean production, area harvested, and yield progress focused on the subset of 12 countries that had on average more than 20,000 ha of soybean cultivation since 2017 (Russia, Ukraine, Italy, Serbia, Romania, France, Croatia, Austria, Hungary, Slovakia, Moldova, and Germany). Data on soybean imports (including intra Europe trade accounting for approximately 25% of total imports) for year 2020 was downloaded from FAOSTATS^7^ under the “Trade”, “Crops and livestock products” domains. Country filter was “Europe > (List)”, and Items were “Soybeans”, “Oil, Soybeans”, and “Cake, Soybeans”. The components “Oil, Soybeans” and “Cake, Soybeans” were converted to soybean seed equivalents using 0.178 and 0.792 oil-to-seed and cake-to-seed ratios, respectively^103^. The list of 39 countries for this dataset included: Albania, Austria, Belarus, Belgium, Bosnia, Bulgaria, Croatia, Czechia, Denmark, Estonia, Finland, France, Germany, Greece, Hungary, Iceland, Ireland, Italy, Latvia, Lithuania, Luxembourg, Malta, Montenegro, Netherlands, North Macedonia, Norway, Poland, Portugal, Moldova, Romania, Russia, Serbia, Slovakia, Slovenia, Spain, Sweden, Switzerland, Ukraine and UK. Data processing and visualisation was performed with the dplyr^104^ and ggplot2 in R^105^. Increase in soybean cultivation was fitted using a Local Polynomial Regression (loess) from the R statistical software. Yield potential and water-limited simulations for each of the 12 selected soybean producing countries was performed with the model described in the next section. Yield trends from FAO data were analysed using linear regression (*lm()* function) in R. We lean toward using a linear regression instead of a non-linear approach to describe yield trends over time because genetic and agronomical improvements can be attributed to the gradual accumulation of small gains and the consistent application of these advancements. Additionally, our focus was to use the most parsimonious statistical model, which, for the reason mentioned above, is the linear regression model^106^.

### Crop modelling and simulations

Soybean performance was simulated by employing a phenological model and physiological model across Europe and surrounding regions with boundaries of a bounding box defined by a minimum and maximum latitude of 35.8 and 61.2 decimal degrees, respectively, and a minimum and maximum longitude of − 10.5 and 48.9 decimal degrees, respectively. The simulations were run on a 0.5º resolution grid (the centroid of which is hereby referred to as a site) for a total of 4,036 sites. Weather inputs of daily minimum temperature, maximum temperature, precipitation, and solar radiation were obtained for each site from The Weather Company’s (an IBM business) Cleaned Historical Observations for 20 years from 1999 to 2018, inclusive. The timing of soybean developmental stages was simulated using an ensemble dynamic phenology model, previously trained on transcontinental data^107^. The publicly available phenology model was modified to include a planting algorithm; planting date for each site and year was determined adaptively based on a rolling mean of growing degree units (GDUs) calculated with base 10 °C and maximum 30 °C. If the seven-day rolling daily mean exceeded 4.4 GDUs, planting would occur, with March 1^st^ as the earliest possible planting date. Emergence was assumed to occur after accumulating 70 GDUs^108^, and therefore planting under conditions that allow emergence within fewer than 16 days. The timing of soybean developmental stages was simulated using an ensemble phenology model with a daily time step^107^. This model ensemble simulates phenological stages based on two physiological models (CROPGRO and SOYDEV) and one machine learning model. Both model components are driven by weather variables such as mean temperature, and daylength. The model was calibrated with ~ 13,500 data points from cultivars maturity group 000 to X across^106^. The phenological stages predicted by the model, emergence, flowering (R1), initiation of seed filling (R5), and physiological maturity (R7), were used to drive a physiology model, also referred to as a crop growth model. The mean absolute error of prediction for the phenological model was ~ 5 days. The public soybean crop growth model, previously parameterized from soybean observations, is based on established principles^109^ using a modified plant leaf area expansion function^110^ and empirical coefficients for the parameters of the expolinear growth equation of the leaf area index (LAI) and leaf partitioning^108^. For each site and year, the model was run for each maturity group from 0000 to III. This analysis did not parameterize neither the phenological or physiological models to the European observations, and so model performance reported is strictly out of sample fits. The quantification of performance is to illustrate the relative potential or plausibility of soybean crops in European geographies.

Four model configurations were run: a “yield potential” simulation calculated outcomes when only solar radiation and temperature were limiting factors (i.e., no water or nutrient limitation), and “rainfed” simulations (i.e. rainfed yield potential) with three different soil moisture initial conditions used to calculate the outcomes under water limitation, generating a total of 2,260,160 unique simulations (4036 sites × 20 years × 7 maturity groups × 4 configurations). For the rainfed simulations, local soil properties (including water holding capacity and wilting point) and initial water availability were obtained using the soil moisture active passive SMAP mission’s v4 L4 land model constants (SPL4SMLM), analysis updates (SPL4SMAU), and geophysical data (SPL4SMGP) products. The SMAP root zone depth of 1 m was used to define maximum rooting depth for soybeans^111^, and a daily average was used for non-SPL4SMLM variables reported at a temporal resolution finer than 1 day^112,113^. Initial conditions corresponding to planting dates outside of the SMAP mission (prior to 2015-03-31) were assumed to be the mean daily value of each site for that calendar day for the six years from 2015 to 2020 (inclusive). Initial plant available water was derived from the estimated SMAP rootzone soil moisture for the planting date (i.e. SPL4SMGP “sm_rootzone”). The ensemble standard deviation of the SMAP root zone soil moisture (i.e. SPL4SMAU “sm_rootzone_ensstd”) was used to estimate the uncertainty in initial soil water conditions, and three initial conditions were used for the rainfed simulations: − 1, 0 and + 1 standard deviation conditions. Given the initial rootzone soil moisture, initial plant available water was defined using the rootzone wilting point (i.e. SPL4SMLM “clsm_wp”); the SMAP holding capacity constant (i.e. SPL4SMLM “clsm_poros”) was used as a basis for determining runoff. Ultimately, each site was characterised by 20 years × 7 maturity groups × 4 water availability configurations (560 simulations per site). In all cases, simulated years were reported as the mean of 20-year weather data).

### Simulation data processing

Outputs were processed to define plausible attainable yields and crop success rates. A given simulation at a site and season (i.e. a given cropping season) was considered a failure if: (1) the mean temperature fell below 0 °C on any day between plant emergence and physiological maturity (R7); (2) the duration between planting and R7 was greater than 170 days following literature^114^ that indicated crops taking longer than 170 days in European environments would be uncommon; or (3) the adaptive planting algorithm never resulted in planting. The yield for a failed site and season was assigned as 0 Mg ha^−1^. For crops that completed their cycle successfully, 7 Mg ha^−1^ was considered as a maximum plausible attainable yield. Whilst yield potentials greater than this are plausible^115^, available European soybean yield trial data (Supplementary Table 2, Supplementary Fig. 1) did not support that yields greater than 7 Mg ha^−1^ would be achievable, and this ceiling was set in the simulations as a conservative maximum yield estimate. Comparing simulated rainfed yield potential to observed rainfed yield trials indicated that simulated potentials were plausible. Rainfed yield potentials of trials from Belgium, Germany, Italy, the Netherlands, Spain, and the United Kingdom were performed without any calibration to the data. The simulations were compared to the observed mean trial yield of each grown maturity, indicating plausible simulated water-limited yield potential (n = 84; Pearson correlation = 0.48; Mean Bias Error = -0.60 Mt ha^−1^; Mean Absolute Percentage Error = 36%; Supplementary Fig. 1).

For each site, three site-level summary statistics were generated: the mean rainfed yield for each of the seven maturity groups (7 variables representing, for each of the 7 maturity groups, the mean of 20 years × 3 initial water conditions); the proportion of years reaching physiological maturity (7 variables representing, for each of the 7 maturity groups, the proportion of non-failures as defined above across 20 years); and the mean planting day of the year (1 variable representing the mean planting day across 20 years, where planting date was identical across maturities). These 15 statistics (7 maturity groups, with their planting date (1 date for all), success rate for each group, and their yield) for each site were used to define regions of similar potential soybean performance across the study area using spatial clustering. Spatial weights were defined based on a 75 km distance band and, following min–max normalization of each variable, and a bottom-up agglomerative hierarchical clustering of individual pixels. The Ward’s method on Euclidean distance was performed with PySal and Scikit-learn to generate 16 clusters^116,117^.

### Environmental savings

To indicate the magnitude of potential environmental savings achievable through enhanced European production of soybean, six simplified scenarios were generated to represent maximum agronomic potential and associated land and GHG emission savings under different contexts of soy production and consumption. These highly stylised scenarios do not represent predictions or forecasts based on current trends, but are designed to indicate the magnitude of possible GHG mitigation associated with possible supply and demand side changes. Two scenarios were created to represent soybean cultivation on 50% of set-aside land, estimated for relevant agroclimatic regions (soybean yields of at least 3 t ha^−1^) based on an average set-aside ratio of 6.1% of arable land in 2022^118^. These scenarios assumed no crop displacement effects, and reflected current (Scenario 1, Sc-1) and potential (Scenario 2, Sc-2) yields from Table 1. Aggregate soybean production was therefore calculated as the sum product of 3.05% of arable areas (50% of 6.1% set aside) for regions supporting at least 3 t ha^−1^ yield (Table 1) multiplied by respective yields in those regions. Another pair of scenarios considered displacement of wheat based on data presented in Table 1. These scenarios assume that 10% of wheat acreage was converted to soy cultivation, in regions where average soybean yields of at least 3 t ha^−1^ could be achieved—at current (Scenario 3, Sc-3) or potential yields (Scenario 4, Sc-4), respectively (same approach to sum product calculation by area per region multiplied by yield per region). Wheat displacement is a simplified yet relevant and conservative assumption for the following reasons: (i) wheat cultivation represents 11–48% of arable areas in regions studied here, proving an obvious target for land sparing and crop system diversification; (ii) large volumes of wheat grain go to animal feed, and could be avoided through the diet change component of scenarios studied here (explicitly modelled in results). The soybean produced was assumed to replace soybean imported into Europe from Brazil, with land and GHG emission intensities taken from the Agribalyse life cycle assessment (LCA)^119^ database accessed via OpenLCA^119^ – a GHG intensity of 1.39 kg CO_2_e per kg soybean is conservative (relatively low) compared with emissions intensities reported in previous studies and Ecoinvent v3.9^120^. The tonnage of wheat displaced was calculated based on the area of soybean cultivation multiplied by an average yield of 7.1 t ha^−1^ based on conventional soft wheat grain production for animal feed in France from Agribalyse. Finally, Sc-3 & 4 were adapted to consider half of the soybean production displacing animal protein, equally split across chicken, pork and milk o the basis of 1:1 protein substitution (Table 1) for chicken and pork, and substitution of 7 kg of cows’ milk with 1 kg soybeans^120^ at current or potential yields (Scenario 5, Sc-5 and Scenario 6, Sc-6, respectively). This substitution provides an estimate of the approximate magnitude of environmental savings that could be leveraged by soy consumption as part of a more radical sustainable food system transformation, as advocated by, *inter alia*, Springmann et al.^121^ Agribalyse inventory data for European production of broilers, pig meat and milk were used to estimate avoided quantities of main feed ingredients (wheat, barley, soybean, maize, rapeseed and sunflower), as well as total avoided production GHG emissions. In turn, inventory data from Agribalyse for European production of the main feed ingredients, excluding soybean, were used to estimate avoided land areas and fertiliser-N applications associated with avoidance of feed production. Avoided soybean production associated with animal protein substitution (50% of new European soybean cultivation) and direct substitution of imported soybean (50% of new European soybean cultivation) was represented based on inventory data for imported Brazilian soybean, as already described. These effects were summed to indicate aggregate GHG, land and fertiliser-N savings associated with animal protein substitution. Finally, land availability will be a major constraining factor for carbon dioxide removal (CDR) activities, such as afforestation, necessary to achieve Paris Agreement commitments^121^. Thus, sparing land from food production could leverage considerable GHG mitigation via CDR – represented here by applying a default carbon sequestration potential value of 3.6 t ha^−1^ year^−1^ proposed by Searchinger et al.^97^ to net land areas spared from animal feed production. These scenarios do not intend to predict the future, but to illustrate the magnitude of environmental savings technically achievable if different agri-food strategies incorporate ambitious expansion of European soybean cultivation. These strategies range from business-as-usual plus simple substitution of imported feed, to more a more radical food system transformation that previous studies have indicated is necessary to respect planetary boundaries^122^.

### Soybean effects on health and industrial properties

Relevant literature was included to identify and synthesize evidence on soybean properties important to human nutrition, health and traits related to food processing in order to compare soy with other protein sources (both plant and animal based). Data on the nutritional value of soy compared to other food products was drawn primarily from the UK Government Composition of Foods Integrated Dataset 2019^123^. This database was selected as it provides data for a wide range of foodstuffs and enables direct comparison across a number of nutritional properties. Additional data for nutritional quality of milks, tofu and maize flour were not available from the database. This data was drawn from other sources as shown in Supplementary Table 1.

### Supplementary Information

Supplementary Information.

## Data Availability

Data utilized in this manuscript can be found at these links: https://ec.europa.eu/eurostat/web/regions/data/database, https://www.fao.org/faostat/en/, https://www.ibm.com/weather, https://smap.jpl.nasa.gov/data/. Data can be provided upon a reasonable request from JLR (jose.rotundo@corteva.com) and MCR (mariana.rufino@tum.de).
