# Supplementary material for: European soybean to benefit people and the environment
Source: Sci Rep. 2024 Mar 31;14:7612. doi: 10.1038/s41598-024-57522-z (PMC10982307; doi:10.1038/s41598-024-57522-z)
Supplement: Supplementary file 1 — Supplementary Information. [file 41598_2024_57522_MOESM1_ESM.docx]

**European soybean to benefit people and the environment**

Jose L. Rotundo et al.

**Supplementary TABLE 1**

Nutritional properties of animal and plant protein sources. Values are provided on an as is basis. Darker, green-shaded cells indicate higher concentrations, blank cells indicate no data available. Most data from Ref.^1^ unless otherwise stated, see footnote.

* Ref.^2^; † Ref. ^3^; ‡ Ref. ^4^; § Ref. ^5^; ‖ Ref.^6^

**Supplementary FIGURE 1**

**
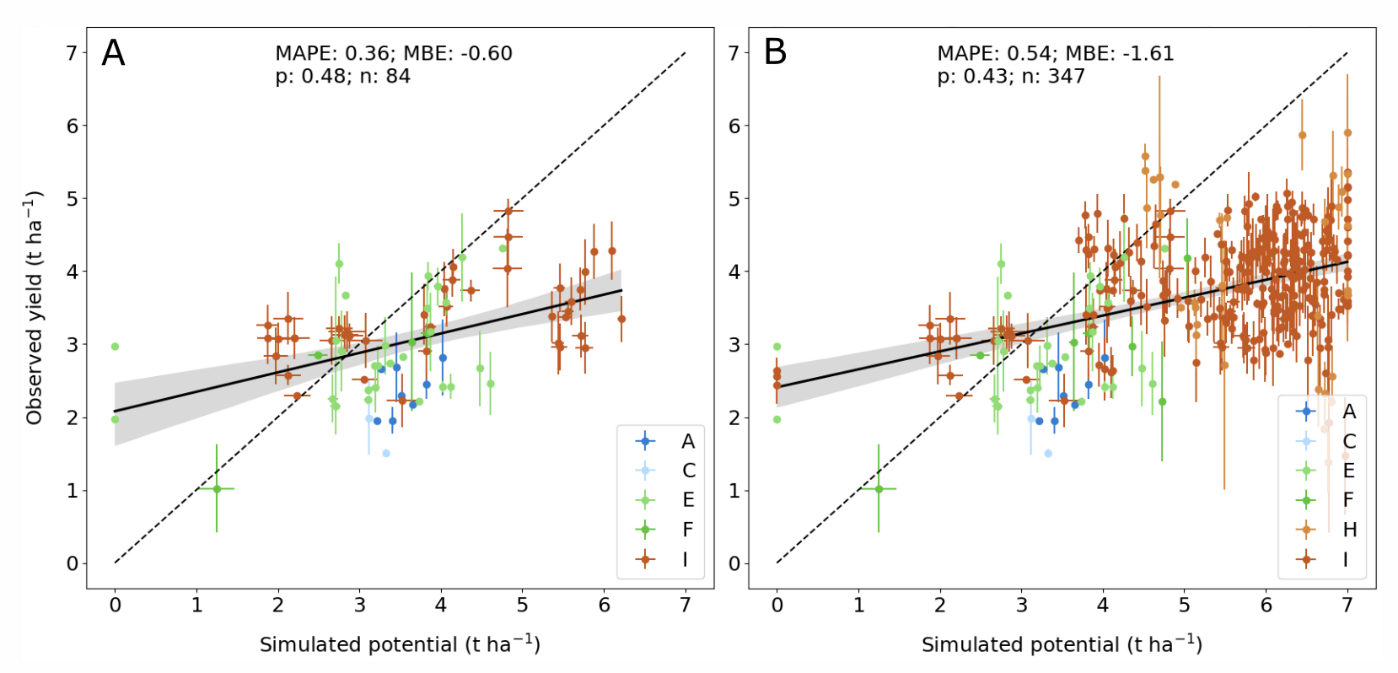
**

Mean yields of observations grouped by site and relative maturity vs mean simulated rainfed yield potentials (for rainfed trials) or yield potentials (for irrigated trials). Observations from trials, colored by regions shown in Figure 2 in which the trials were located. Vertical and horizontal bars represent the standard deviation of the observations grouped by site and relative maturity (for the y axis) and grouped by the simulations of relative maturity and different initial soil water conditions (for the x axis; only for rainfed yield potentials), respectively. (A) observations from rainfed trials and simulated water-limited yield potentials and only, (B) observations from both irrigated and rainfed trials, and simulations of yield potentials and rainfed yield potentials. MAPE is the mean absolute prediction error; MBE is the mean bias error; p is the Pearson correlation coefficient.

**Supplementary Table 2.**

Experimental observations are defined as the combination of site (latitude and longitude), planting date (day of the year and year), and cultivars (relative maturity). These observations were used to evaluate model performance as depicted in Supplementary Fig. 1.

| Country | Experiment observations | |
| --- | --- | --- |
|  | Rainfed | Rainfed + Irrigated |
| Italy | 42 | 270 |
| Spain | n/a | 35 |
| Belgium | 22 | 22 |
| Netherlands | 14 | 14 |
| Germany | 4 | 4 |
| UK | 2 | 2 |
| Total | 84 | 347 |

**REFERENCES**

1 Phe. (Public Health England, 2021).

2 Usda. (US Department of Agriculture, Agricultural Research Service, Nutrient Data Laboratory, 2016).

3 Correia, P., Soares, A. & Brites, C. M. Quality characteristics of maize flours and breads. *Int J Food Eng* **2**, 113-118, doi:10.18178/ijfe.2.2.113-118 (2016).

4 European Food Safety, A. (2021).

5 Chalupa-Krebzdak, S., Long, C. J. & Bohrer, B. M. Nutrient density and nutritional value of milk and plant-based milk alternatives. *Int. Dairy J.* **87**, 84-92, doi:10.1016/j.idairyj.2018.07.018 (2018).

6 Singhal, S., Baker, R. D. & Baker, S. S. A comparison of the nutritional value of cow's milk and nondairy beverages. *J Pediatr Gastr Nutr* **64**, 799-805, doi:10.1097/mpg.0000000000001380 (2017).
